# Ssanghwa-tang, an oriental herbal cocktail, exerts anti-melanogenic activity by suppression of the p38 MAPK and PKA signaling pathways in B16F10 cells

**DOI:** 10.1186/1472-6882-13-214

**Published:** 2013-08-28

**Authors:** Aeyung Kim, Nam-Hui Yim, Minju Im, Young Pil Jung, Chun Liang, Won-Kyung Cho, Jin Yeul Ma

**Affiliations:** 1 Korean Medicine (KM)-Based Herbal Drug Development Group, Korea Institute of Oriental Medicine (KIOM), 483 Expo-ro, Yuseong-gu, Daejeon 305-811, Republic of Korea

**Keywords:** Ssanghwa-tang, Melanogenesis, p38 MAPK, PKA, MITF, Tyrosinase, B16F10 cells

## Abstract

**Background:**

Ssanghwa-tang (SHT) is a widely used medication for the treatment of fatigue, pain, inflammation, hypothermia, erectile dysfunction, cancer, and osteoporosis in Asia, however, role of SHT on the melanin synthesis has not been checked previously. Thus, the present study was designed to determine the effect of SHT on α-melanocyte stimulating hormone (α-MSH)-induced melanogensis and its mechanisms of action in murine B16F10 melanoma cells.

**Method:**

Cellular melanin content and tyrosinase activity in murine B16F10 melanoma cells were determined after α-MSH stimulation with or without pre-treatment of SHT at the concentration of 250 and 500 μg/ml. Expression level of tyrosinase, tyrosinase-related protein 1 (TRP-1), TRP-2, microphthalmia-associated transcription factor (MITF), and activation of c-AMP-dependent protein kinase (PKA), c-AMP-related element binding protein (CREB), and mitogen-activated protein kinases (MAPKs) were examined by Western blot analysis.

**Results:**

SHT significantly inhibited α-MSH-induced melanin synthesis and tyrosinase activity, and also decreased α-MSH-induced expression of MITF, tyrosinase, and TRP-1. In addition, SHT remarkably suppressed tyrosinase, CRE, and MITF luciferase reporter activity in a resting state as well as in α-MSH-stimulating condition. Phosphorylation of p38 MAPK by α-MSH stimulation was efficiently blocked by SHT pre-treatment. Moreover, SHT as an herbal cocktail showed synergistic anti-melanogenic effect compared with that of each single constituent herb.

**Conclusion:**

SHT efficiently inhibited c-AMP-induced melanin synthesis in B16F10 cells *via* suppression of PKA and p38 MAPK signaling pathways and subsequently decreased the level of CREB phosphorylation, MITF, and melanogenic enzymes. These results indicate that SHT may be useful as herbal medicine for treating hyperpigmentation and cosmetics as a skin-whitening agent.

## Background

Melanin, the main component determining the color of skin, hair, and eyes in mammals, is synthesized by melanocytes within specialized organelles named melanosomes, which are then transferred to adjacent keratinocytes through the dendritic tips of melanocytes, resulting in the distribution throughout the epidermis [1]. Melanin synthesis is mainly regulated by tyrosinase gene family, including tyrosinase, tyrosinase-related protein 1 (TRP-1), and TRP-2. Tyrosinase plays an essential role in the modulation of melanin synthesis by catalyzing the hydroxylation of L-tyrosine into 3,4-dihydroxyphenylalanine (DOPA) and the further oxidation of DOPA into dopaquinone. TRP-2, dopachrome tautomerase, catalyzes the rearrangement of dopachrome into 5,6-dihydroxyindole-2-carboxylic acid (DHICA), whereas TRP-1 oxidizes DHICA into a carboxylated indole-quinone [2]. Melanogenesis can be triggered by a large number of effectors, including ultraviolet B radiation [3] and c-AMP-inducing agents (e.g. α-MSH, forskolin) [4]. c-AMP activates c-AMP-dependent protein kinase (PKA) and c-AMP-related element binding protein (CREB) transcription factor, and increases the expression of microphthalmia-associated transcription factor (MITF), a master regulator of melanocyte differentiation, pigmentation, proliferation, and survival [5]. Since genes encoding tyrosinase and TRP-1 are under transcriptional control of the MITF, substances capable of inhibiting MITF expression and activity could substantially down-regulate melanogenesis.

Under normal physiological conditions, pigmentation has a beneficial effect on the photo-protection of human skin against harmful UV injury, and plays an important evolutionary role in camouflage and animal mimicry [6]. Meanwhile, abnormal skin hyper-pigmentation such as melasma, freckles, senile lentigines, chloasma, and melanoderma incurred by inflammation including eczema, allergic contact dermatitis, and irritant contact dermatitis, causes serious and distressing skin problems [7]. Depigmentation can be achieved by down-regulation of the expression and activity of tyrosinase, TRP-1, and TRP-2, by regulation of the uptake and distribution of melanosomes in keratinocytes, and by degradation of melanin and melanosome. Due to the pivotal role of tyrosinase in melanogenesis, identification of tyrosinase inhibitors is the most potent approach for the development of cosmetic products and medicinal drug treating abnormal skin pigmentation [8]. Commercially available natural melanin synthesis inhibitors including arbutin, kojic acid, and stilbene often cause undesirable side-effects. For example, kojic acid, a naturally occurring hydrophilic fungal derivative evolved from certain species of *Acetobacter*, *Aspergillus*, and *Penicillium*, displayed the potential to cause contact dermatitis and erythema [9]. Therefore, novel skin-whitening agents with more potent efficacy but less adverse effect are necessary for cosmetic and medicinal purposes.

Ssanghwa-tang (SHT), a traditional herbal medicine, has been widely used for thousands of years in Korea, China, and Japan to treat infirmity, relieve fatigue, and facilitate recovery after an illness. SHT has been depicted in the Dongui Bogam, a Korean book followed by the royal physician published in 1613, and reported to have several pharmacological effects including analgesic, hepatoprotective, anti-inflammatory, and anti-osteoporosis effects [10,11]. SHT consists of 9 medicinal herbs, including *Paeonia lactiflora*, *Angelica gigas*, *Astragalus membranaceus*, *Cnidium officinale*, *Rehmannia glutinosa*, *Glycyrrhiza glabra*, *Cinnamomum cassia*, *Zingiber officinale*, and *Zizyphus jujube*. Some respective single herbs in SHT have already proved to inhibit melanogenesis through inhibition of trysosinase activity [12]. However anti-melanogenic effect of SHT and its fundamental mechanism of action are not clearly elucidated. Therefore, in the present study, we investigated the effects of SHT on the melanogenesis in B16F10 cells under c-AMP-stimulating condition, and examined detailed mechanism of its anti-melanogenic activity.

## Methods

### Cell lines

The murine melanoma B16F10 cell line was purchased from American Type Culture Collection (ATCC, Manassas, VA) and maintained as a monolayer culture in Dulbecco’s Modified Eagle Medium (DMEM; Lonza, Walkersville, MD) supplemented with 10% (v/v) heat-inactivated fetal bovine serum (FBS; GIBCO/Invitrogen, Carlsbad, CA), 100 units/ml penicillin, and 100 μg/ml streptomycin (Welgene, Korea) at 37°C in a humidified 5% CO_2_ incubator. For the preparation of murine hepatocytes, 6–8 weeks old female ICR mouse were purchased from Nara Bio animal center (Nara Biotech, Korea), and housed under standard conditions at a temperature of 24 ± 1°C and humidity of 55 ± 5%. The experimental procedures were approved by Korea Institute of Oriental Medicine Care and Use Committee with a reference number 12–093 and carried out in accordance with the Korea Institute of Oriental Medicine Care Committee Guidelines. Hepatocytes were isolated using a perfusion system with some modification [13]. Cells suspended in the William’s E medium containing 10% FBS, 100 IU/ml insulin, 2 mM L-glutamine, 15 mM HEPES, 100 units/ml penicillin, and 100 μg/ml streptomycin were seeded on the culture plate coated with 10% gelatin/phosphate buffered saline (PBS), and incubated at 37°C in a humidified 5% CO_2_ incubator.

### Antibodies and reagents

Alpha-melanocyte stimulating hormone (α-MSH), forskolin, synthetic melanin, mushroom tyrosinase, _L_-3,4-Dihydroxyphenylalanine (_L_-DOPA), and 3-(4,5-Dimethyl-2-thiazolyl)-2,5-diphenyltetrazolium bromide (MTT) were purchased from Sigma Chemical Co. (St Louis, MO, USA). Antibodies against tyrosinase, TRP-1, TRP-2, MITF, PKA, and phospho-PKA (Thr198) were obtained from Santa Cruz Biotechnology Inc. (Santa Cruz, CA, USA). Anti-CREB, anti-phospho-CREB (Ser133), anti-p38, anti-phospho-p38 (Thr180/Tyr182), anti-extracellular signal-related kinase1/2 (ERK), anti-phospho-ERK (Thr202/Tyr204), anti-c-Jun-N-terminal kinase (JNK), anti-phopsho-JNK (Thr183/Tyr185), and anti-tubulin antibodies were purchased from Cell Signaling Technology (Danvers, MA, USA). All of the other chemicals and solvents used were analytical grade.

### Preparation of Ssanghwa-tang (SHT)

SHT is composed of 9 medicinal herbs, the root of *Paeonia lactiflora* (28%), root of *Angelica gigas* (11.2%), root of *Astragalus membranaceus* (11.2%), root of *Cnidium officinale* (11.2%), preparata of root of *Rehmannia glutinosa* (11.2%), root of *Glycyrrhiza glabra* (8.4%), bark of *Cinnamomum cassia* (8.4%), root of *Zingiber officinale* (4.4%), and fruit of *Zizyphus jujube* (6.0%), which were purchased from Korea Medicinal Herbs Association (Yeongcheon, Korea). Identification of all herbs was confirmed by Prof. KiHwan Bae of the College of Pharmacy, Chungnam National University (Daejeon, Korea), and all voucher specimens were deposited in the herbal band in Korea Institute of Oriental Medicine (KIOM, Korea). SHT formula were extracted in distilled water by heating for 3 h at 115°C in an extractor (Cosmos-600 Extractor, Gyeonseo Co., Inchon, Korea) and then filtered using standard testing sieves (150 μm, Retsch, Haan, Germany). The freeze-dried SHT extract was dissolved in PBS, filtered (0.45 μm), and then kept at 4°C prior to use.

### Cell viability assay

Cells, seeded at a density of 5 × 10^3^ cells/well in 96-well culture plates, were cultured overnight and then treated with various concentrations of SHT (25, 50, 100, 250, 500, 1000, and 2000 μg/ml) or single herbal extract for the 48 h. After cells were incubated with 10 μl of MTT solution (5 mg/ml in PBS) for 4 h, the formazan precipitates were dissolved by dimethyl sulfoxide (DMSO) and then absorbance was measured at 570 nm with Infinite® M200 microplate reader (TECAN Group Ltd. Switzerland). Cell viability was presented as the percentage of viable cells compared with untreated, control cells.

### Measurement of cellular melanin contents

Cellular melanin content was measured as described previously [14]. Briefly, B16F10 cells seeded at a density of 3 × 10^5^ cells on the 100 mm culture dishes were pre-treated with 250 and 500 μg/ml of SHT for 12 h, and then stimulated with 1 μM of α-MSH for additional 36 h. After harvest of cells, equal number of cells (1 × 10^7^ cells/sample) were dissolved in 100 μl of 1 N NaOH/10% DMSO for 1 h at 80°C, and solubilized melanin was measured at 475 nm using Infinite® M200 microplate reader. Relative melanin content compared with untreated “control” cells was calculated from a standard curve using synthetic melanin.

### Measurement of tyrosinase activity

B16F10 cells seeded in 6-well plates (1 × 10^5^ cells/well) were pre-treated with 250 and 500 μg/ml of SHT for 12 h, and then further incubated with 1 μM of α-MSH for 36 h. For the measurement of cellular tyrosinase activity, the cells were washed with ice-cold PBS and then lysed with 1% Triton X-100 in PBS by repeated freezing/thawing. Each lysate was centrifuged at 12000 rpm for 15 min at 4°C to obtain a supernatant as a source of tyrosinase, and then determined for protein concentration. The reaction mixture containing same amount of supernatant (or mushroom tyrosinase) compensated with 50 mM phosphate buffer (pH 6.8) up to 90 μl and 10 μl of 10 mM _L_-DOPA as a substrate for tyrosinase was incubated at 37°C in a 96-well plate. Following incubation, dopachrome formation from _L_-DOPA was monitored by measuring the absorbance at 475 nm using Infinite® M200 microplate reader, and relative tyrosinase activity was calculated from that of standard mushroom tyrosinase. Relative tyrosinase activity was expressed as a percentage compared with untreated, control cells.

### Luciferase reporter assay

For the analysis of tyrosinase, CRE, and MITF promoter activity, semi-confluent cells grown in 12-well culture plates were transiently transfected with each luciferase reporter plasmid (pTyrosinase-luc, pCRE-luc, and pMITF-luc) and Renilla luciferase plasmid using TransIT®-2020 Transfection reagent (Mirus Bio LLC., Madison, WI) according to the manufacturer’s instructions. After incubation, cells were lysed in passive lysis buffer, and the luciferase activities were measured by luminescence microplate reader set (TriStar LB 941, Berthold Technologies GmbH and Co. KG, Bad Wildbad, Germany) using dual luciferase reporter assay system according to the manufacturer’s instructions (Promega, Madison, WI).

### Western blot analysis

After washing cells twice with PBS, whole cell lysates were extracted in M-PER mammalian protein extraction reagent (Thermo Scientific, Rockford, IL) by centrifugation (12000 *g* × 15 min, 4°C), and the protein concentration was determined using Bicinchoninic Acid (BCA) Kit (Sigma). Total protein (40–80 μg) was separated by electrophoresis on 10–12% SDS-polyacrylamide gels, and transferred to Immobilon®-P PVDF transfer membrane (Millipore, Bedford, MA). After immunoblotting, proteins were visualized using a PowerOpti-ECL Western blotting detection reagent (Animal Gentetics, Inc. Korea) and an ImageQuant LAS 4000 mini (GE Healthcare, Piscataway, NJ). Equal amount of proteins was analyzed by Western blotting using tubulin as a loading control and band intensities were quantified using ImageJ software (National Institutes of Health, USA).

### Preparation of standard solutions and SHT and analytical chromatic conditions

For the qualitative analysis, 10 standard compounds, paeoniflorin, liquiritin, nodakenin, benzoic acid, nodakenetin, decursinol, cinnamyl alcohol, cinnam aldehyde, decursin, and decursinal angelate, were prepared by dissolving in 100% methanol as described previously [15]. Analytical SHT sample was prepared by dissolving powder in 100% H_2_O at a concentration of 40 mg/mL followed by filtration through a 0.45 μm filter. The main components profile of SHT was analyzed at the 254 nm UV wavelength using the Elite Lachrom HPLC system (Hitachi High-Technologies Co., Tokyo, Japan) consisting of pump (L-2130), auto-sampler (L-2200), column oven (L-2350), and diode array UV/VIS detector (L-2455). System control and data analyses were executed by EZchrom Elite software (verson 3.3.1a) system. The chromatographic separation was conducted with RS-tech C_18_ column (Optimapak C_18_, 4.6 × 250 mm, 5 μm, Daejeon, Korea) at 40°C and the injection volume was 10 μl. The mobile phase was a gradient elution of 1% acetic acid and acetonitrile at a flow rate of 1 ml/min, commencing with 5% acetonitrile for 5 min, linear gradient to 100% acetonitrile was applied over 70 min, and then maintained at 100% for 10 min.

### Statistical analysis

Data are presented as the mean ± SD values of at least 3 independent experiments, unless otherwise specified. Statistical significance was analyzed by the two-tailed student’s *t*-test in Sigma Plot 8.0 software (SPSS Inc., Chicago, IL) and a *P* value of less than 0.05 was considered statistically significant.

## Results

### SHT at non-cytotoxic concentrations inhibits melanin synthesis in B16F10 cells

**Figure 1:**
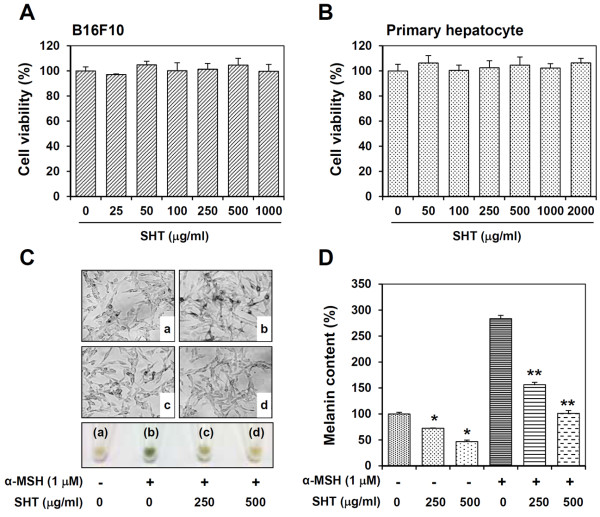
**Effects of SHT on the cell viability and melanin production in B16F10 cells.** B16F10 cells **(A)** and murine primary hepatocytes **(B)** were incubated with indicated concentrations of SHT for 48 h, and then the cell viability was determined by MTT assay. **(C)** B16F10 cells were pre-incubated with or without 250 and 500 μg/ml SHT for 12 h, stimulated with 1 μM of α-MSH for additional 36 h, and then observed for the accumulation of melanin in the microscopic image or pigmentation of cell pellets (2 × 10^6^ cells). Image magnification, ×200. **(D)** After harvesting cells, cellular melanin content in the same number of cells was determined. Each percentage value was calculated with respect to that of untreated “control” cells as 100%. Data are expressed as the mean ± SD of three independent experiments. *p < 0.05 *vs* control, **p < 0.05 *vs* α-MSH-treated cells.

To exclude the possibility that the inhibitory effect of SHT on melanin synthesis was due to cytotoxicity, we determined whether SHT is toxic to B16F10 cells using a MTT assay. SHT did not significantly affect cell morphology and did not cause any noticeable cytotoxicity during incubation at concentrations up to 1000 μg/ml for 48 h. Thus, in all subsequent experiments, SHT was used at 250 or 500 μg/ml (Figure 1A). In addition, SHT did not cause cytotoxicity in murine primary hepatocytes, even after incubation with 2000 μg/ml for 48 h (Figure 1B), suggesting that SHT is non-toxic at a wide range of concentrations. Treatment with α-melanocyte stimulating hormone (MSH), which stimulates cAMP production, caused a 280% accumulation of melanin in cells, resulting in a black-pigmented cell pellet as reported previously. Pre-treatment with SHT remarkably blocked α-MSH-induced melanin production and black pigmentation in a dose-dependent manner (Figure 1C). At baseline, B16F10 cells produced a substantial amount of melanin during incubation, and SHT treatment at 250 or 500 μg/ml reduced melanin production to 70 or 45% of untreated control levels, respectively (Figure 1D). Thus, in cells pre-treated with SHT at a dose of 500 μg/ml, the increase in α-MSH-induced melanin remained relatively low, and the melanin level was similar to that of untreated control cells, suggesting that SHT completely blocks α-MSH-mediated melanogenesis.

### SHT suppresses tyrosinase activity, CRE, and MITF promoter activity in B16F10 cells

**Figure 2:**
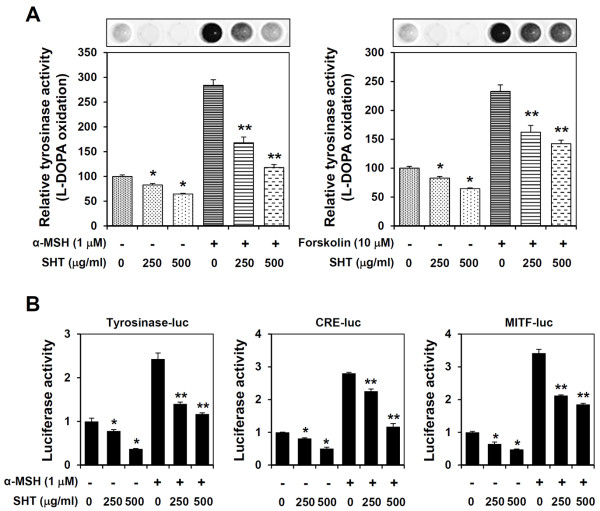
**Effects of SHT on the cellular tyrosinase activity and melanogenesis-related promoter activation. (A)** B16F10 cells were pre-incubated with or without 250 and 500 μg/ml SHT for 12 h, stimulated with 1 μM of α-MSH for additional 36 h, and then cellular tyrosinase activity was determined by measuring the formation of dopachrome from _L_-DOPA. Visible dopachrome formation was shown in upper panel and relative tyrosinase activity compared with untreated “control” cells was calculated. **(B)** To determine the effect of SHT on the tyrosinase, CRE and MITF promoter activation, B16F10 cells were transfected with pTyrosinase-luc, pCRE-luc, and pMITF-luc reporter DNA, and incubated for 24 h in the presence or absence of α-MSH and SHT. The data are representative of three independent experiments carried out in triplicate, and expressed as mean ± SD. *p < 0.05 *vs* control, **p < 0.05 *vs* α-MSH-treated cells.

To elucidate the inhibitory mechanism of melanogenesis by SHT, we assessed tyrosinase activity in cell lysates by measuring _L_-DOPA oxidation. In resting B16F10 cells, treatment with 250 and 500 μg/ml of SHT decreased tyrosinase activity by 17% and 36%, respectively (Figure 2A). The involvement of the protein kinase A (PKA) pathway was investigated by treating cells with the cAMP inducer α-MSH (1 μM) or forskolin (10 μM), which significantly increased tyrosinase activity by 285 or 230%, respectively. These increases were dose-dependently inhibited by SHT pre-treatment: 500 μg/ml SHT decreased α-MSH- or forskolin-induced tyrosinase activity by 60 or 40%, respectively (Figure 2A). Increases in cAMP levels upregulate the activity of the MITF promoter through activation of cAMP response element (CRE)-binding transcription factor, and MITF binds to and activates the tyrosinase promoter [5]. We performed luciferase reporter assays in B16F10 cells transfected with the tyrosinase, CRE, or MITF promoter to examine the effect of SHT on promoter activity. As shown in Figure 2B, luciferase activity was elevated to 2.5–3.5 times the baseline level by α-MSH treatment, and SHT treatment dose-dependently suppressed tyrosinase, CRE, and MITF luciferase reporter activity in untreated cells and in cells stimulated with α-MSH. In α-MSH-stimulated cells, SHT (500 μg/ml) decreased tyrosinase, CRE, and MITF promoter activities by 52, 58, and 48%, respectively, compared with the activities in untreated control cells. These results indicate that SHT functionally inhibits melanogenesis by inactivating CRE and MITF promoter activity to suppress tyrosinase activity.

### SHT inhibits the expression of melanogenic enzymes in B16F10 cells and downregulates phosphorylation of p38 MAPK

**Figure 3:**
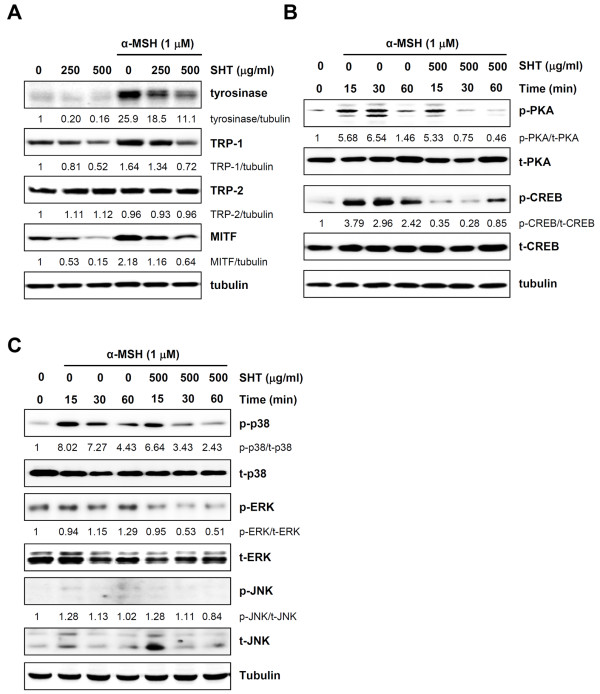
**Effects of SHT on the expression levels of melanogenic proteins, p-PKA, PKA, p-CREB, CREB, p-MAPKs, and MAPKs after stimulation with α-MSH in B16F10 cells. (A)** B16F10 cells were pre-incubated with or without 250 and 500 μg/ml SHT for 12 h, and then stimulated with 1 μM of α-MSH for 36 h. After harvest of cells, lysates were prepared and subjected to Western blot analysis with the anti-tyrosinase, anti-TRP-1, anti-TRP-2, or anti-MITF antibody. **(B-C)** Cells were pre-incubated with or without 500 μg/ml SHT for 12 h, and then further stimulated with 1 μM of α-MSH for 15, 30, and 60 min. Changes in the level of p-PKA and p-CREB **(B)** and in the level of p-MAPKs including p-p38, p-ERK, and p-JNK **(C)** were examined by Western blot analysis followed by quantitation with ImageJ. Data are representative of two independent experiments.

As melanin synthesis is principally regulated by the tyrosinase gene family, including tyrosinase, TRP-1, TRP-2, and MITF, the effect of SHT on the expression of these proteins was determined by Western blot analysis. In resting B16F10 cells, SHT (500 μg/ml) significantly reduced tyrosinase, TRP-1, and MITF expression levels by 84, 48, and 85%, respectively (Figure 3A). In cells stimulated with α-MSH, the tyrosinase, TRP-1, and MITF expression levels were significantly increased, although the change in TRP-2 expression was insignificant. Pre-treatment with SHT (500 μg/ml) prominently suppressed the α-MSH-induced increase in tyrosinase, TRP-1, and MITF expression by 58, 55, and 70%, respectively, compared with expression in untreated control cells (Figure 3A).

To further investigate whether SHT can regulate the PKA pathway, the effect of SHT on cAMP-induced PKA and CREB phosphorylation was determined by Western blot analysis (Figure 3B). Phosphorylated PKA (p-PKA) and CREB (p-CREB) were barely detectable in resting B16F10 cells. Upon exposure to α-MSH for 15 min, the levels increased significantly by 5.7-fold and 3.8-fold, respectively, compared with the levels in untreated cells. In contrast, pre-treatment with SHT significantly reduced p-PKA by 6, 89, and 69% at 15, 30, and 60 min after α-MSH stimulation, respectively, and p-CREB by 90, 91, and 65% at the respective time points compared with levels in cells treated with α-MSH alone. There was no change in total PKA or CREB expression. These results demonstrate that SHT treatment can regulate events upstream of cAMP-induced melanogenesis and can inhibit melanin synthesis through downregulation of major melanogenic enzymes.

The mitogen-activated protein kinase (MAPK) family proteins, including p38, ERK, and JNK, are known to play critical roles in melanogenesis. For example, the ERK and/or JNK/SAPK pathways cause downregulation of melanin synthesis. In contrast, the phosphorylation of p38 can activate MITF expression, which in turn transcriptionally upregulates the expression of melanogenic enzymes such as tyrosinase, TRP-1, and TRP-2, eventually inducing melanin production [16,17]. To examine the underlying molecular mechanisms involved in the hypopigmentation property of SHT, MAPK signal transduction was detected by Western blot analysis. As shown in Figure 3C, the phosphorylation of p38 MAPK was significantly elevated 8-fold following 15 min of α-MSH stimulation in B16F10 cells and remained elevated for up to 60 min; no remarkable increase in the phosphorylation of ERK or JNK was observed. Pre-treatment with SHT significantly decreased the phosphorylation of p38 MAPK by 17, 53, and 45% following 15, 30, and 60 min of stimulation with α-MSH, respectively, compared with levels in SHT-untreated cells. SHT pre-treatment did not significantly influence the phosphorylation of ERK or JNK, suggesting that ERK and JNK do not contribute to the anti-melanogenic activity of SHT. These results indicate that the suppression of p38 MAPK phosphorylation coupled with reduced expression of MITF and melanogenic enzymes contributes to the anti-melanogenic effect of SHT in B16F10 cells.

### SHT, as a cocktail of single medicinal herbs, has a synergistic anti-melanogenic effect

Many individual medicinal herbs have greater pharmacological efficacy when used as part of an herbal cocktail. To evaluate the possible synergistic effect of SHT, the anti-melanogenic activity of SHT was compared with the individual activity of nine different herbs. Cells were treated for 48 h with each herb at its concentration in 500 μg/ml SHT (Table 1). At these concentrations, single herbs showed no cytotoxicity in B16F10 cells, similar to the SHT herbal cocktail. At baseline, most single herbs did not exhibit anti-tyrosinase activity, except for *Z. jujube* (21%), and some herbs increased tyrosinase activity. Upon α-MSH stimulation, *Z. officinale* and *Z. jujube* inhibited tyrosinase activity by 28 and 14%, respectively, but none of the nine single herbs in SHT possessed potent anti-melanogenic activity. The sum of the individual activities of all nine herbs was only 65% of the activity of SHT, suggesting combinatorial and synergistic effects among multiple herbs in SHT.

**Table 1:** Effects of single medicinal herb of SHT on the cell viability and tyrosinase activity

**Treatment**	**Concentration (μg/ml)**	**Viability (%)**	**Tyrosinase activity (%)**	
			**No stimulation**	**α-MSH stimulation**
Control	0	100.0 ± 2.3	100.0 ± 4.6	287.7 ± 7.1
*P. lactiflora*	140	104.5 ± 1.8	130.8 ± 7.6	322.5 ± 8.2
*A. gigas*	56	101.2 ± 0.6	126.6 ± 2.5	306.8 ± 3.3
*A. membranaceus*	56	108.2 ± 1.3	152.9 ± 8.1	370.6 ± 5.0
*C. officinale*	56	100.0 ± 1.1	96.5 ± 3.2	276.1 ± 1.1
*R. glutinosa*	56	108.5 ± 0.8	101.7 ± 4.9	282.9 ± 1.1
*G. glabra*	41.9	102.3 ± 0.3	122.5 ± 1.1	374.6 ± 9.0
*C. cassia*	22.3	106.8 ± 0.6	140.4 ± 6.9	338.8 ± 3.6
*Z. officinale*	41.9	104.3 ± 1.4	86.3 ± 9.2	207.6 ± 5.5**
*Z. jujube*	29.9	100.5 ± 0.2	78.9 ± 3.6*	246.2 ± 2.8**
SHT	500	104.4 ± 2.4	58.9 ± 5.5*	100.5 ± 3.1**

The data are representative of two independent experiments carried out in triplicate, and expressed as mean ± SD. *p < 0.05 *vs* control, **p < 0.05 *vs* α-MSH-treated cells.

### HPLC analysis of SHT

**Figure 4:**
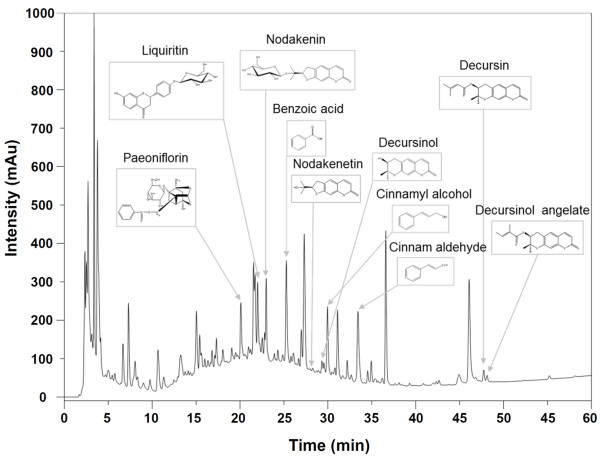
**HPLC chromatogram of SHT at a wavelength of 254 nm and chemical structure of each compound were depicted.** Paeoniflorin, liquiritin, nodakenin, benzoic acid, nodakenetin, decursinol, cinnamyl alcohol, cinnam aldehyde, decursin, and decursinol angelate were identified.

To identify the ingredients of SHT responsible for the inhibition of melanin synthesis in B16F10 cells, HPLC analysis was performed to identify 10 marker components in SHT and the representative chromatogram at a wavelength of 254 nm was shown in Figure 4. Ten components in SHT were detected at the same retention times (*t*_R_) and UV spectrum acquired from HPLC analysis of standard components as follows: paeoniflorin, *t*_R_ 20.12 min; liquiritin, *t*_R_ 22.06 min; nodakenin, *t*_R_ 23.01 min; benzoic acid, *t*_R_ 25.29 min; nodakenetin, *t*_R_ 28.35 min; decursinol, *t*_R_ 29.39 min; cinnamyl alcohol, *t*_R_ 30.00 min; cinnam aldehyde, *t*_R_ 33.47 min; decursin, *t*_R_ 47.81 min; decursinol angelate, *t*_R_ 48.21 min. The content of each compound in SHT was identified as follows: paeoniflorin, 1.136 μM; liquiritin, 0.122 μM; nodakenin, 0.130 μM; benzoic acid, 0.415 μM; nodakenetin, 0.003 μM; decursinol, 0.010 μM; cinnamyl alcohol, 0.032 μM; cinnamaldehyde, 0.033 μM; decursin, 0.009 μM; decursinol angelate, 0.010 μM [15].

## Discussion

SHT is a traditional herbal formula widely prescribed to improve general health and to alleviate symptoms of congestion, pain, and seizure. In recent studies by our group, SHT significantly reduced receptor activator of nuclear factor κB ligand-induced tartrate-resistant acid phosphatase activity and multinucleated osteoclast formation in RAW264.7 cells, with no cellular cytotoxicity. In addition, SHT administration blocked ovariectomy-induced bone loss through an inhibitory effect on osteoclast differentiation [10]. In the present study, we demonstrated the anti-melanogenic activity of SHT and the underlying mechanisms of action in B16F10 cells. SHT at non-cytotoxic concentrations dramatically reduced the melanin content of untreated, as well as α-MSH-stimulated, cells via suppression of tyrosinase activity and MITF expression (Figure 2 and Figure 3A).

Previous studies have demonstrated that p38 MAPK is a major intracellular signaling molecule essential for pigmentation; activation of the p38 MAPK pathway increases melanin synthesis by upregulating MITF expression to promote tyrosinase transcription. [18] Recent studies have reported that the Chinese herbal formula of San-bai-tang and the aqueous fraction from the *Cuscuta japonica* seed inhibit melanogenesis by downregulating the expression of MITF and melanogenic enzymes through the suppression of p38 MAPK [19,20]. Thus, the inhibition of the p38 MAPK cascade may be critical to downregulate melanogenesis. This notion is supported by the present data demonstrating that SHT blocks α-MSH-induced p38 MAPK and PKA phosphorylation, which are critical to MITF expression (Figure 3B, C).

Oriental herbal medicines have long been used for treating a wide range of human diseases and improving physical strength. A number of traditional herbal formulas of Korean, Chinese, and Japanese medicines are multi-herb mixtures. Herbal cocktails containing myriad phytochemicals simultaneously affect multiple biological and pathological processes via synergistic and reciprocal actions. Appropriately formulated herbal cocktails may act in concert to amplify the therapeutic efficacy of each single herb, thereby maximizing therapeutic efficacy while minimizing adverse effects [21,22]. These combined actions are known as pharmacological or pharmaceutical combinatorial effects. As an example, Juzen-taiho-to, which is composed of 10 different herbs, positively modulates systemic immune function of T and B cells, macrophages, NK cells, and the intestinal immune system, whereas any of the 10 single herbs in the formula fail to show similar activity [23]. In the present study, we evaluated the potential combinatorial effects of herbs in SHT on the inhibition of melanin synthesis. Our results revealed that the single herbs in SHT, with the exception of *Z. officinale* and *Z. jujube,* have no anti-melanogenic activities, whereas SHT exerts synergistic anti-melanogenic activity without unwanted side effects such as cytotoxicity (Table 1). Several herbs in SHT, including *A. gigas*, *C. officinale*, *Z. officinale*, and *Z. jujube*, have been reported to modulate melanogenesis; however, the effective doses were much higher and potentially cytotoxic compared with the doses used in our experiments. The methanol extract of *C. officinale* exhibited tyrosinase inhibitory activity with an IC_50_ of 9.6 mg/ml [24], whereas the ethanol extracts of *Z. officinale* and *Z. jujube* inhibited tyrosinase activity by approximately 40% at 330 μg/ml and by 20.3% at 4 mg/ml, respectively [25,26]. Although the ethanol extract of *A. gigas* (AGE) at 5–30 μg/ml remarkably inhibited melanin synthesis in a dose-dependent manner, AGE at 20, 25, and 30 μg/ml reduced cell viability to 90, 80, and 60%, respectively, compared with untreated control cells [12]. In contrast, SHT is a comparatively safe formulation; at concentrations up to 2000 μg/ml, it did not cause cytotoxicity in murine melanoma cells or normal hepatocytes (Figure 1A, B).

Ten marker components in SHT, including paeoniflorin, liquiritin, nodakenin, benzoic acid, nodakenetin, decursinol, cinnamyl alcohol, cinnamaldehyde, decursin, and decursinol angelate, were identified by HPLC analysis (Figure 4) and the most abundant was paeoniflorin (1.136 μM). The extract of the *Paeonia lactiflora* flower, with paeoniflorin as the primary ingredient, has a whitening effect [27]. In addition, some compounds have been reported to retain potent inhibitory effects on melanin synthesis [28,29], supporting the anti-melanogenic effect of SHT. Our group recently analyzed the compositional changes of fermented SHT compared with conventional SHT by HPLC-DAD, MS, and NMR [15]. Upon fermentation of herbs, some glycosides are deglycosylated and reduced in size, which makes them more effective by increasing their absorption and bioavailability in the body [30]. For this reason, a comparative study between conventional and bioconverted herbal formulas (*e.g.*, SHT and fermented SHT) on melanogenesis is a topic of great interest.

## Conclusions

In summary, our finding clearly demonstrated the anti-melanogenic activity of SHT *via* suppression of PKA and CREB activation both in a resting state and in α-MSH-stimulating state in B16F10 cells. Moreover, SHT blocked α-MSH-induced p38 MAPK phosphorylation as well, consequently reduced MITF expression and tyrosinase activity essential for melanin synthesis. Collectively, these results suggest that SHT may be a useful as herbal medicine for treating abnormal skin hyperpigmentation and cosmetics as a skin-whitening agent.

## Abbreviations

α-MSH: α-melanocyte stimulating hormone; TRP: Tyrosinase-related protein; MITF: Microphthalmia-associated transcription factor; PKA: c-AMP-dependent protein kinase A; CREB: c-AMP-related element binding protein; MAPK: Mitogen-activated protein kinase; HPLC: High performance liquid chromatography.

## Competing interests

All authors are in agreement with the content of the manuscript and declare no financial or intellectual conflicts of interests regarding this study.

## Authors’ contributions

AYK conceived of the study, carried out the assay for melanin synthesis, tyrosinase activity, and promoter activity, and signaling analysis. NHY participated in cell culture and determination of cytotoxicity. MJI and YPJ participated in the preparation of SHT and single herbs extract and statistical analysis. CL carried out HPLC analysis. JYM and WKC participated in the design and coordination of study. AYK drafted manuscript. All authors read and approved the final manuscript.

## Pre-publication history

The pre-publication history for this paper can be accessed here:

http://www.biomedcentral.com/1472-6882/13/214/prepub
